# Investigation of the Definition of De Novo Oligometastatic Nasopharyngeal Carcinoma: A Retrospective Study

**DOI:** 10.1155/2021/9977455

**Published:** 2021-09-15

**Authors:** Hongmei Wang, Fang He, Xuejun Wang, Haoyun Tao, Zhihao Huang, Yawei Yuan, Weijun Zhang

**Affiliations:** ^1^Department of Radiation Oncology, Affiliated Cancer Hospital & Institute of Guangzhou Medical University, Guangzhou, Guangdong, China; ^2^Department of Radiation Oncology, People's Hospital of Zhongshan City, Zhongshan, Guangdong, China

## Abstract

**Background:**

The prognosis of metastatic nasopharyngeal carcinoma (mNPC) is highly heterogeneous. As a special stage of distant metastasis of mNPC, quite a few oligometastatic NPC (omNPC) patients can still achieve a long-term survival after treatment. However, there is no uniform standard for the definition of omNPC until now.

**Methods:**

We retrospectively analyzed the survival data of 191 patients with de novo mNPC at the Affiliated Cancer Hospital and Institute of Guangzhou Medical University between 2010 and 2017 and specifically analyzed the clinical outcomes associated with the number of metastatic organs/lesions and tried to find a cohort with relatively better prognosis to define as omNPC.

**Results:**

The median overall survival (OS) of the entire group of patients was 21.5 months (95% CI 15.0–28.0), and the 1-year, 2-year, and 3-year OS rates were 72.2%, 46.1%, and 34.3%, respectively. Multiple-organ metastases (*P* < 0.001) and >5 metastatic lesions (*P* < 0.001) were adverse influencing factors of prognosis, and the number of metastatic lesions (*P* < 0.001) was the independent factor influencing the prognosis of de novo mNPC. The overall survival (OS) and progression-free survival (PFS) of patients with ≤5 metastatic lesions were significantly better than those of patients with >5 metastatic lesions.

**Conclusion:**

Patients with ≤5 metastatic lesions presented a better survival, and this criterion may be a definition standard for the de novo omNPC.

## 1. Introduction

Nasopharyngeal carcinoma (NPC) is a head and neck malignant tumor with a high incidence in South China and Southeast Asia, and the main pathological type is squamous cell carcinoma [1]. Compared with other head and neck malignant tumors, NPC has a higher tendency of lymph node metastasis and distant metastasis at an early stage [2, 3]. With the wide application of intensity-modulated radiotherapy (IMRT) and positron emission tomography- (PET-) computed tomography (CT) in tumor diagnosis and staging, the early diagnosis rate and overall survival (OS) rate of NPC have been further improved for the 5-year OS reaches up to 85% for early and locally advanced NPC after treatment [4, 5], but the prognosis of mNPC is still highly heterogeneous [6, 7]. Approximately 4%–10% of NPC patients have distant metastasis at initial diagnosis (stage IVB, American Joint Committee on Cancer (AJCC), 8th edition) [7, 8], and mNPC is generally considered incurable and has a poor overall prognosis, with a median OS of approximately 20 months [6]. However, some patients with limited disease can still achieve long-term survival after traditional palliative treatment combined with locoregional cancer treatments such as radiotherapy and surgical excision, and some even achieved clinical cure [9, 10].

The concept of tumor oligometastasis was first proposed in 1995 and amended in 2011 [11] and generally defined as a special tumor transfer condition between local invasion and polymetastatic, and at this time, tumors have a low disease burden and a weak invasive ability because of the limited number and size of metastatic lesions, so the tumor could be cured [12]. This concept has long been proposed and widely used in the field of nonsmall cell lung cancer (NSCLC), colorectal cancer, and breast cancer [13–16]. However, the definition criteria for oligometastasis are not exactly the same for different tumor types, and most studies defined oligometastasis as ≤5 metastases. The guidelines for metastatic colorectal cancer published by the European Society of Oncology (ESMO) in 2016 pointed out that oligometastatic colorectal cancer usually refers to a disease state with metastatic organs ≤2 and number of metastases ≤5 [16]. In 2017, the ESMO proposed that oligometastatic breast cancer is defined as a tumor burden state in which the number and size of metastases are limited, the number of metastases is ≤5, and the metastases are not necessarily limited to the same organ [17]. But in the field of NPC, there are relatively fewer studies on oligometastasis, and it is uncertain whether these definition criteria are equally applicable to NPC.

In the latest Union for International Cancer Control (UICC)/AJCC 8th TNM staging system for NPC, the M1 stage (stage IVB) covers a variety of conditions from single metastasis in one organ to multiple-organ metastasis. This cannot distinguish and screen out patients with greater therapeutic significant and cannot well predict the prognosis, so it is necessary to further stratify the M1 stage. Meanwhile, there is still no consensus on the specific definition of omNPC, and the influence of the number of metastatic organs and lesions on the prognosis of NPC has not yet been clearly evaluated. Based on these backgrounds, this study explored the definition of de novo omNPC and the related prognostic factors, hoping to provide a reference for the clinical formulation of reasonable individualized treatment options.

## 2. Materials and Methods

The participants in this study were retrospectively recruited from the Affiliated Cancer Hospital and Institute of Guangzhou Medical University between January 2010 and July 2017. A total of 191 patients were included, and the inclusion criteria were as follows: de novo mNPC and received at least one cycle of systemic chemotherapy and/or local radiotherapy. Patients with other malignancies, did not receive antitumor therapy, and those missing clinical/survival data (for survival analysis) were excluded. This study was approved by the Ethics Committee of the Affiliated Cancer Hospital and Institute of Guangzhou Medical University, which waived the requirement for written informed consent because of the retrospective nature of the study.

All patients completed a comprehensive inspection and evaluation before starting treatment, which includes a complete physical examination and medical history data recorded, and completed nasopharyngoscopy, electrocardiogram, chest X-ray or chest CT, abdominal ultrasound or CT, nasopharyngeal and neck magnetic resonance imaging (MRI), bone scan, and relevant blood examination. Some patients also completed PET-CT. All patients underwent T staging and N staging again on the basis of the UICC/AJCC TNM classification system (8th edition, 2017). The metastatic organs and metastatic lesions were reviewed and determined jointly by two senior physicians (including one radiologist).

### 2.1. Treatments

All patients received at least 1 cycle of palliative chemotherapy. The chemotherapy regimens were mainly platinum-containing doublet or triplet chemotherapy regimens, and single-agent chemotherapy was adopted for patients which were considered intolerant to combination chemotherapy. The first-line chemotherapy regimens mainly included TP (docetaxel plus cisplatin), PF (fluorouracil plus cisplatin), GP (gemcitabine plus cisplatin), and TPF (docetaxel plus cisplatin plus fluorouracil), and the single-drug chemotherapy regimen was docetaxel. Chemotherapy regimens used for the analysis were the first-line regimens implemented from the beginning of treatment to disease progression, while the second-line and third-line chemotherapy regimens were not included.

Local radiotherapy was added to patients who achieved complete response (CR) or partial response (PR) by imaging examination after chemotherapy and in an acceptable performance status or in patients with severe symptoms caused by the primary tumor. The delineation of the target volumes of 3D conformal RT and intensity-modulated radiotherapy (IMRT) was majorly defined according to reports ICRU 50 and ICRU 62. The primary tumor GTV includes retropharyngeal nodes, GTVnx, and gross cervical lymph nodes (GTVnd). The high-risk clinical tumor volume (CTV1) was then defined with the concept of “5 + 5 mm expansion” margin from GTV to delineate CTV1. On the basis of CTV1, the target volume of CTV2 was expanded by 5–10 mm plus positive lymph nodes area and lymph node drainage area requiring preventive irradiation. The planned target volume (PTV) was expanded by 0.5 cm circumferentially and 0.3 cm posteriorly automatically by the planning system mainly according to uncertainty factors such as positioning error per week. A total of 149 (78.0%) patients received local radiotherapy to the primary tumor and regional lymph nodes including 77.2% of the patients (115 of 149) treated with IMRT techniques and 22.8% (34 of 149) treated with 3D conformal RT. The median dose was 70 Gy (range, 40.0–70.0 Gy). Furthermore, 44 patients also received local therapy to the metastases, including radiofrequency ablation of liver or lung metastases, radiotherapy for liver metastasis, and palliative radiotherapy for bone metastases.

The tumor response was assessed by reviewing the results of physical examinations and radiological investigations according to the WHO criteria and generally conducted after every 2 cycles of chemotherapy or 3 months after finishing the whole treatment.

### 2.2. Statistical Method

The main prognostic indicators of this study included OS and PFS. OS was calculated from the date of diagnosis to the date of death or the last follow-up, and PFS was defined as the interval between the initial diagnosis and the imaging examination indicating tumor recurrence or progression, death due to any cause or the last follow-up. All the analyses were performed with SPSS version 25.0 software. The Kaplan–Meier method was used to calculate the survival rate and estimate the median survival time, and the significance of differences between different subgroups was compared by using the log-rank test. The covariates that were identified by the univariate analysis as significantly associated (*P* < 0.05) with prognosis were included in the Cox proportional hazards regression model for the multivariate analysis and were subsequently selected by forward conditional selection, and then, the survival curve was drawn. Two-sided *P* values <0.05 were considered statistically significant.

## 3. Results

**Figure 1:**
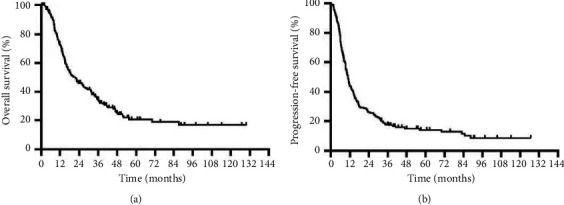
Kaplan–Meier plot showing OS and PFS for the entire cohort.

The characteristics of the 191 patients are given in Table 1. Among the 191 patients, 153 (80.1%) were male and 38 (19.9%) were female. The median age was 49 years (range: 19–79 years). Overall, 126 (66.0%) patients had single-organ metastasis, 65 (34.0%) had multiple-organ metastasis, 61 (31.9%) had 1–3 metastatic lesions, 33 (17.3%) had 4-5 metastatic lesions, and 97 (50.8%) had >5 metastatic lesions. At the cutoff of July 2020, the median follow-up time was 18.0 months (range 0.6–129.7 months). The median OS for the entire cohort was 21.5 months, and the median PFS was 10.8 months. The 1-year, 2-year, and 3-year OS rates of the entire cohort were 72.2%, 46.1%, and 34.3%, respectively. The survival curves for the entire cohort are shown in Figure 1.

**Figure 2:**
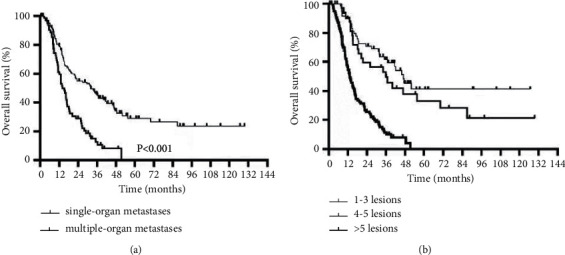
OS curves of different subgroups of metastatic organs (a) and metastatic lesions (b).

In the univariate analysis, Karnofsky performance status (KPS) score <80 (*P* < 0.001), N2-3 (*P*=0.007), multiple-organ metastasis (*P* < 0.001), metastatic lesions >5 (*P* < 0.001), lung metastasis (*P*=0.026), liver metastasis (*P* < 0.001), no radiotherapy to the primary tumor (*P* < 0.001), radiotherapy dose <70 Gy (*P* < 0.001), and first-line chemotherapy cycles <6 (*P*=0.045) were adverse prognostic factors for the OS of de novo mNPC patients. Among them, the prognosis of patients with multiple-organ metastasis was significantly worse than that of patients with single-organ metastasis (median OS 14.0 vs 31.9 months, *P* < 0.001) (Figure 2(a)), and the 3-year OS was 12.9%, much lower than 45.1% in patients with single-organ metastasis. The prognosis of patients with >5 metastatic lesions was significantly worse than that of patients with ≤5 metastatic lesions (median OS 18.9 vs 51.2 months, *P* < 0.001), and the 3-year OS was 11.2%, which was much lower than the 57.2% for patients with ≤5 metastatic lesions. Moreover, among patients with ≤5 metastatic lesions, there was no significant difference in OS between the 1-3 and 4-5 metastatic lesion subgroups (*P*=0.244) (Figure 2(b)).

The multivariate analysis of prognostic factors in patients with de novo mNPC showed that the KPS score (*P*=0.024), number of metastases (*P* < 0.001), whether nasopharyngeal radiotherapy was received (*P* < 0.001), radiotherapy dose (*P*=0.001), and the number of first-line chemotherapy cycles (*P*=0.013) were independent prognostic factors of de novo mNPC. The results of the univariate and multivariate analyses are summarized in Tables 1 and 2. Thus, we trend to support the hypothesis that ≤5 metastatic lesions might be a feasible standard for defining the de novo omNPC.

**Figure 3:**
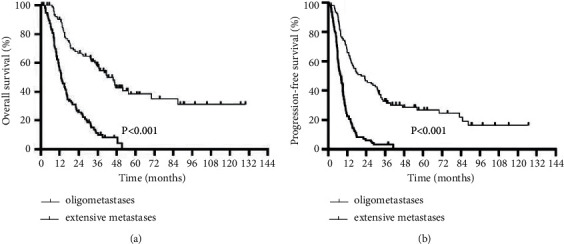
Kaplan–Meier plot comparing the OS and PFS between the oligometastatic group and extensive metastasis group.

According to this definition, this study included 94 patients with oligometastasis and 97 patients with extensive metastasis. The baseline data of these two groups are given in Supplementary Table 1. Among the 94 patients with oligometastasis, 19 patients have received local treatment for distant metastases, including 2 cases of liver radiofrequency ablation, 1 case of local radiotherapy for lung metastases, and 4 cases of local radiotherapy for metastatic lymph nodes. The median OS was 62.5 months (95% CI 36.20–57.19) in the oligometastasis group and 17.9 months (95% CI 11.02–15.79) in the extensive metastasis group (HR = 0.257, 95% CI 0.18–0.37; *P* < 0.0001). The 1-year, 2-year, and 3-year OS rates in the oligometastasis group were 90.1%, 66.7%, and 57.2%, respectively. There were 15 patients whose survival time was longer than 5 years, and the longest survival time even reached to 129.7 months. The 1-year, 2-year, and 3-year OS rates in the extensive metastasis group were 54.5%, 25.7%, and 11.2%, respectively, and none of the patients reached to a survival time of five years. Similarly, the median PFS in the oligometastasis group was significantly better than the extensive metastasis group (42.3 months and 9.8 months, respectively) (*P* < 0.0001), and the risk of disease progression was reduced by 71% (HR = 0.29; 95% CI 0.20–0.40). The survival curves are shown in Figure 3.

## 4. Discussion

In the latest UICC/AJCC 8th edition TNM staging system for NPC, stage IVB includes a variety of metastases from one single lesion to polymetastatic metastases. Although these different forms belong to the M1 stage, the therapeutic response is not always completely consistent in patients with different metastatic burdens, and correspondingly, the prognosis will be variously different. Oligometastasis is a special subgroup of the M1 stage and occurs in the early stages of clinical tumor metastasis course, and this subgroup is characterized by the limited number of metastatic lesions, the limited burden of metastatic lesions, and the relatively small potential to develop to polymetastatic disease, which makes the local treatment of metastatic lesions significant and makes it possible to cure the tumor [12]. In fact, in the stage of oligometastasis, effective systemic treatment combined with local treatment of metastatic lesions, such as surgical resection, stereotactic radiotherapy, and radiofrequency ablation, can prolong the overall survival time and even achieve complete remission and has already been demonstrated in a variety of solid tumors, such as NSCLC, colorectal cancer, and breast cancer [15–17]. Both the ESMO and NCCN guidelines have made recommendations for the management of oligometastasis in these tumors. However, there are few recommended guidelines on omNPC.

To the best of our knowledge, this study is one of the few studies in investigating omNPC. Among previous studies, the criteria for the definition of omNPC were highly diverse, which made it difficult to compare the results of different studies and draw precisely conclusions [18, 19]. Meanwhile, some studies incorporated both primary metastasis patients and posttreatment metastasis patients, and their treatment strategies were different, and certainly, the prognosis could not be generalized. In this study, we analyzed the survival prognosis of patients with different numbers of metastatic organs and lesions and found that patients with ≤5 metastatic lesions achieved a superior clinical outcome in both OS and PFS than those patients with >5 metastatic lesions. Patients with single organ metastasis were found to have a significantly longer OS compared with multiple-organ metastasis, the number of organ metastasis was not the independent prognostic factor of the entire cohort, which may indicate that when the tumor burden is limited, the number of involved organs was not the most important prognostic factor. This conclusion is consistent with some previous studies, and the rationality of this conclusion and the reliability of the results of this study were confirmed again based on this retrospective analysis.

Locoregional radiotherapy to the primary site is receiving increasing attention in advanced NPC. Numerous retrospective analyses have indicated that the combination of primary site radiotherapy can obviously improve the prognosis of de novo mNPC, such as improving the local control rates and reducing the possibility of further extensive metastasis, and especially in oligometastatic disease, may achieve a radical cure [7, 18, 20, 21]. A phase III randomized multicenter clinical trial that investigated the efficacy and safety of locoregional radiotherapy in chemotherapy-sensitive de novo mNPC proved that locoregional radiotherapy added to chemotherapy will significantly improve the OS of patients [22]. In our study, patients in the entire cohort who received radiotherapy to the primary tumor has achieved a significantly better prognosis compared with those patients who did not receive radiotherapy, and the vast majority of patients (93.6%) with ≤5 metastatic lesions have received radiotherapy to the primary tumor, which indicates that intensifying the local radiotherapy of the primary tumor might improve survival of patients with de novo mNPC, especially in patients with lower tumor burden.

Different from polymetastatic patients, some oligometastatic patients may reach the cure standard if strengthening the treatment to metastatic sites on the basis of controlling the primary sites [23, 24]. Li et al. [23] analyzed 328 patients with NPC with liver metastasis by the propensity score matching analysis and found that systemic chemotherapy plus CT-guided radiofrequency ablation of the liver can significantly improve OS compared with chemotherapy alone, especially in patients with fewer metastases in whom the benefit is more obvious. The patients' 3-year and 5-year OS increased from 25.3% and 3.9% to 41.3% and 29.5%, and the 3-year and 5-year PFS increased from 5.6% and 5.6% to 22.0% and 8.4%, respectively [23]. In addition, some retrospective studies have found that adopting different treatment methods for metastatic sites, such as radiotherapy for bone metastasis, radiotherapy for distant metastatic lymph nodes, and surgical resection or stereotactic radiotherapy for lung metastasis, can bring survival benefits to patients to various degrees [25–27]. However, we failed to get the same conclusion in this study. This may have resulted from the small sample size and the time of treatment of metastatic sites. Some patients underwent treatment for the metastases lesions only when the lesions developed symptoms such as compression and pain, rather than treating the metastases in the oligometastatic stage. Therefore, the intervention time for the treatment of metastases is an urgent issue that needs to be discussed and solved.

Nasopharyngeal carcinoma is a tumor which is sensitive to chemotherapy. Though lacking of high-level evidence to support the benefit of systematic chemotherapy for advanced NPC is compared with optimal supportive treatment, systematic chemotherapy is still the cornerstone of comprehensive treatment for mNPC and has been proven to be significantly correlated with the prognosis by the plentiful retrospective analysis [20, 28, 29]. Zhang et al. [28] conducted a multicenter prospective study to compare the safety and efficacy of the GP regimen and PF regimen in recurrent or metastatic NPC, and the results showed that the patients obtained a significant PFS benefit from the GP regimen compared with the PF regimen. Meanwhile, it was observed that the PFS of patients with oligometastasis was 7.3 months which was higher than the 6.9 months of patients with multiple metastases in the GP regimen group. In our study, most patients enrolled had experienced recurrence or metastasis after treatment, and the findings failed to directly prove the superiority of the GP regimen in primary omNPC. Therefore, more prospective studies on omNPC should be carried out in the future.

In summary, this study retrospectively analyzed the clinical data of 191 patients and tried to seek out a feasible definition of de novo omNPC, which was aiming to better stratify the stage M1 NPC patients and to help clinicians better distinguish patients with greater therapeutic needs and provide a reference for making reasonable individualized treatment strategies. At present, the understanding of omNPC is still insufficient and needs further research to identify specific molecular biomarkers as well as the safely and effective intervention methods and timing. These are also the new challenges that the multidisciplinary treatment modes for mNPC are going to face.

There are some limitations in this study. First, the study did not restrict the volume of the oligometastatic lesions, which may ignore the influence of tumor burden on prognosis. Another limitation is lacking of data on important biomarkers, such as plasma EBV-DNA and lactate dehydrogenase before and during treatment. Finally, as a survival analysis study, the sample size of this study was small, so the results and conclusions need to be further verified by high-quality prospective studies.

## 5. Conclusion

According to the definition of ≤5 metastatic lesions, de novo mNPC patients with great therapeutic need can be better distinguished out and given a more active individualized treatment regimen.

## Figures and Tables

**Table 1:** The clinical baseline data and univariate survival analysis of 191 patients.

Factor	Cases (%)	Median OS (month)	HR (95% CI)	*P* value
Sex				
Male	153 (80.1)	41.8		0.563
Female	38 (19.9)	35.7	1.13 (0.75–1.72)	

Age				
≤49	102 (53.4)	41.2		0.853
>49	89 (46.6)	41.1	1.03 (0.74–1.45)	

Pathological type				
WHO type I/II	14 (7.3)	41.4		0.198
WHO type III	177 (92.7)	19.2	1.46 (0.82–2.59)	

KPS				
<80	50 (26.2)	15.9		<0.001
≥80	141 (73.8)	48.8	0.35 (0.24–0.51)	

*T* stage				
1-2	38 (19.9)	51.9		0.440
3-4	153 (80.1)	34.0	1.19 (0.77–1.84)	

*N* stage				
0-1	27 (14.1)	64.7		0.007
2-3	164 (85.9)	36.4	2.07 (1.21–3.57)	

Number of metastatic organs				
Single	126 (66.0)	51.2		<0.001
Multiple	65 (34.0)	18.9	2.41 (1.69–3.45)	

Number of metastases				
1–3	61 (31.9)	68.5		
4-5	33 (17.3)	53.7	1.40 (0.80–2.43)	0.244
>5	97 (50.8)	17.9	4.37 (2.79–6.86)	<0.001

Bone metastasis				
Yes	124 (64.9)	43.2		0.747
No	67 (35.1)	28.5	0.94 (0.66–1.35)	

Lung metastasis				
Yes	52 (27.2)	23.7		0.026
No	139 (72.8)	45.7	0.66 (0.45–0.95)	

Liver metastasis				
Yes	70 (36.6)	20.7		<0.001
No	121 (63.4)	50.4	0.49 (0.35–0.70)	

Nasopharyngeal radiotherapy				
Yes	149 (78.0)	47.4		<0.001
No	42 (22.0)	16.1	2.80 (1.89–4.14)	

Dose at primary tumor				
<70 Gy	25 (16.8)	18.4		<0.001
≥70 Gy	124 (83.2)	51.1	0.38 (0.23–0.63)	

Platinum-containing chemotherapy				
Doublet	128 (69.6)	39.4		0.162
Triplet	56 (30.4)	39.6	0.76 (0.51–1.12)	

Cycles of first-line chemotherapy				
<6	124 (64.9)	37.3		0.045
≥6	67 (35.1)	48.2	0.69 (0.48–0.99)	

Metastasis treatment				
Yes	44 (23.0)	29.9		0.552
No	147 (77.0)	43.7	0.89 (0.61–1.31)	

**Table 2:** Multivariate survival analysis of 191 patients.

Factor	Cases (%)	Median OS (month)	HR (95% CI)	*P* value
KPS				
<80	50 (26.2)	11.6		0.024
≥80	141 (73.8)	31.2	1.77 (1.08–2.91)	

Number of metastases				
1-3	61 (31.9)	46.9	—	—
4-5	33 (17.3)	35.9	1.36 (0.76–2.45)	0.297
>5	97 (50.8)	13.0	3.99 (2.36–6.78)	<0.001

Nasopharyngeal radiotherapy				
No	42 (22.0)	10.3		<0.001
Yes	149 (78.0)	27.9	0.02 (0.01–0.16)	

Dose				
<70 Gy	25 (16.8)	14.4		0.001
≥70 Gy	124 (83.2)	31.9	0.42 (0.25–0.71)	

Cycles of first-line chemotherapy				
<6	124 (64.9)	15.6		0.013
≥6	67 (35.1)	31.8	0.57 (0.36–0.89)	

## Data Availability

All data generated or analyzed during this study are included within this article.

## Abbreviations

NPC: Nasopharyngeal carcinoma
mNPC: Metastatic nasopharyngeal carcinoma
omNPC: Oligometastatic nasopharyngeal carcinoma
OS: Overall survival
PFS: Progression-free survival
KPS: Karnofsky
IMRT: Intensity-modulated radiotherapy.

## Supplementary Materials

Supplementary Materials We provide the clinical data of patients with oligometastatic and extensive metastases in Supplementary Table 1. And clinical characteristics of 94 oligometastatic patients are given in Supplementary Table 2.
