# Supplementary material for: Investigation of the Definition of De Novo Oligometastatic Nasopharyngeal Carcinoma: A Retrospective Study
Source: J Oncol. 2021 Sep 15;2021:9977455. doi: 10.1155/2021/9977455 (PMC8460388; doi:10.1155/2021/9977455)
Supplement: Supplementary Materials — We provide the clinical data of patients with oligometastatic and extensive metastases in Supplementary Table 1. And clinical characteristics of 94 oligometastatic patients are given in Supplementary Table 2. [file 9977455.f1.doc]

[Supplementary](javascript:;) [table](javascript:;) 1. Clinical characteristics of patients with oligometastatic and extensive metastasis

| Factor | oligometastatic  (%) | extensive metastasis  (%) | P value |
| --- | --- | --- | --- |
| Sex |  |  |  |
| Male | 78 (83.0) | 75（77.3） | 0.327 |
| Female | 16 (17.0) | 22（22.7） |  |
| Age |  |  |  |
| ≤49 | 45 (47.9) | 57（58.8） | 0.131 |
| >49 | 49 (52.1) | 40（41.2） |  |
| Pathological type |  |  |  |
| WHO typeⅠ/Ⅱ | 9 (9.5) | 5（51.5） | 0.241 |
| WHO type Ⅲ | 85 (90.5) | 92（48.5） |  |
| KPS |  |  |  |
| <80 | 83 (88.3) | 58（59.8） | ＜0.001 |
| ≥80 | 11(11.7) | 39（40.2） |  |
| T stage |  |  |  |
| 1-2 | 19 (20.2) | 19（19.6） | 0.914 |
| 3-4 | 75 (79.8) | 78（80.4） |  |
| N stage |  |  |  |
| 0-1 | 20 (21.3) | 41（42.3） | 0.002 |
| 2-3 | 74 (78.7) | 56（57.7） |  |
| Number of metastatic  organs |  |  |  |
| Single | 85 (90.4) | 41 (42.3) | ＜0.001 |
| Multiple | 9 (9.6) | 56 (57.7) |  |
| Bone metastasis |  |  |  |
| Yes | 58 (61.7) | 66 (68.0) | 0.359 |
| No | 36 (38.3) | 31 (32.0) |  |
| Lung metastasis |  |  |  |
| Yes | 15 (16.0) | 37 (38.1) | 0.001 |
| No | 79 (84.0) | 60 (61.9) |  |
| Liver metastasis |  |  |  |
| Yes | 18 (19.1) | 52 (53.6) | ＜0.001 |
| No | 76 (80.9) | 45 (46.4) |  |
| Nasopharyngeal  radiotherapy |  |  |  |
| Yes | 88 (93.6) | 61 (62.9) | ＜0.001 |
| No | 6 (6.4) | 36 (31.7) |  |
| Dose at primary tumor |  |  |  |
| < 70 gy | 10 (11.4) | 15（24.6） | 0.034 |
| ≥70 gy | 78 (88.6) | 46（75.4） |  |
| Platinum-containing chemotherapy |  |  |  |
| Doublet | 66 (75.2) | 62（66.7） | 0.388 |
| Triplet | 25 (24.8) | 31（33.3） |  |
| Cycles of first-line chemotherapy |  |  |  |
| <6 | 64 (68.1) | 60（61.9） | 0.367 |
| ≥6 | 30 (31.9) | 37（38.1） |  |
| Metastasis treatment |  |  |  |
| Yes | 71 (75.5) | 76 (78.4) | 0.644 |
| No | 23 (24.5) | 21 (21.6) |  |

[Supplementary](javascript:;) [table](javascript:;) 2. Clinical characteristics of 94 patients with oligometastatic

| Factor | Cases (%) | P value |
| --- | --- | --- |
| Sex |  |  |
| Male | 78 | 0.532 |
| Female | 16 |  |
| Age |  |  |
| ≤50 | 48 | 0.284 |
| >50 | 46 |  |
| Pathological type |  |  |
| WHO typeⅠ/Ⅱ | 10 | 0.447 |
| WHO type Ⅲ | 84 |  |
| KPS |  |  |
| <80 | 11 | 0.01 |
| ≥80 | 83 |  |
| T stage |  |  |
| 1-2 | 19 | 0.041 |
| 3-4 | 75 |  |
| N stage |  |  |
| 0-1 | 20 | 0.148 |
| 2-3 | 74 |  |
| Bone metastasis |  |  |
| Yes | 58 | 0.099 |
| No | 36 |  |
| Lung metastasis |  |  |
| Yes | 15 | 0.476 |
| No | 79 |  |
| Liver metastasis |  |  |
| Yes | 18 | 0.156 |
| No | 76 |  |
| Nasopharyngeal  radiotherapy |  |  |
| Yes | 88 | 0.064 |
| No | 6 |  |
| Dose at primary tumor |  |  |
| < 70gy | 10 | 0.094 |
| ≥70 gy | 78 |  |
| Platinum-containing chemotherapy |  |  |
| Doublet | 66 | 0.214 |
| Triplet | 25 |  |
| Cycles of first-line chemotherapy |  |  |
| <6 | 64 | 0.109 |
| ≥6 | 30 |  |
| Metastasis treatment |  |  |
| Yes | 23 | 0.121 |
| No | 71 |  |
